# Low-grade undifferentiated sarcoma with *MEIS1::NCOA2*-rearrangement primary to the lung: a case report

**DOI:** 10.1186/s13000-024-01484-3

**Published:** 2024-04-27

**Authors:** Zachary Coty-Fattal, Bianca Carter, Michael J. Volek, Farres Obeidin

**Affiliations:** Department of Pathology, Northwestern University Feinberg School of Medicine, Chicago, IL USA

**Keywords:** Translocation, *MEIS1:NCOA*, Low-grade, Undifferentiated, Sarcoma

## Abstract

**Background:**

*MEIS1::NCOA2* is a rare fusion gene that has been recently described in a subset of spindle cell rhabdomyosarcomas and multiple low-grade undifferentiated spindle cell sarcomas predominantly arising in the genitourinary and gynecologic tracts with no specific line of differentiation. We present the first documented case of this neoplasm arising as a lung primary tumor.

**Case Presentation:**

A 74-year-old woman with a 40-year smoking history presented with a 2.1 × 1.7 cm lung nodule discovered on computed tomography (CT) scan. A biopsy and subsequent lobe resection were performed, as well as an extensive metastatic work up, which revealed no additional masses. No specific line of differentiation was found by immunohistochemical staining, and an RNA-based fusion panel revealed a *MEIS1::NCOA2* fusion, at which point a diagnosis of Low-Grade Undifferentiated Sarcoma with *MEIS1::NCOA2*-Rearrangement was rendered.

**Conclusions:**

This report represents the first diagnosis of this tumor primary to the lung, and provides additional insight into the origin and localization of these rare tumors.

## Background

*MEIS1::NCOA2* is a rare fusion gene that has been recently described in a subset of spindle cell rhabdomyosarcomas and multiple low-grade undifferentiated spindle cell sarcomas predominantly arising in the genitourinary and gynecologic tracts [1–3]. We present a case of a 74-year-old woman who presented with a 2.1 cm right upper lobe lung nodule. The lesion was composed of whorls and fascicles of monomorphic and infiltrative spindled cells with minimal mitotic activity and no necrosis. Immunohistochemical staining showed no specific line of differentiation, but next generation sequencing revealed an *MEIS1:NCOA2* fusion. Ultimately, given a completely negative metastatic workup, a diagnosis of lung primary low-grade undifferentiated spindle cell sarcoma with *MEIS1::NCOA2* fusion was rendered. To our knowledge, no other lung primary tumors of this type have been reported.

## Case presentation

**Fig. 1:**
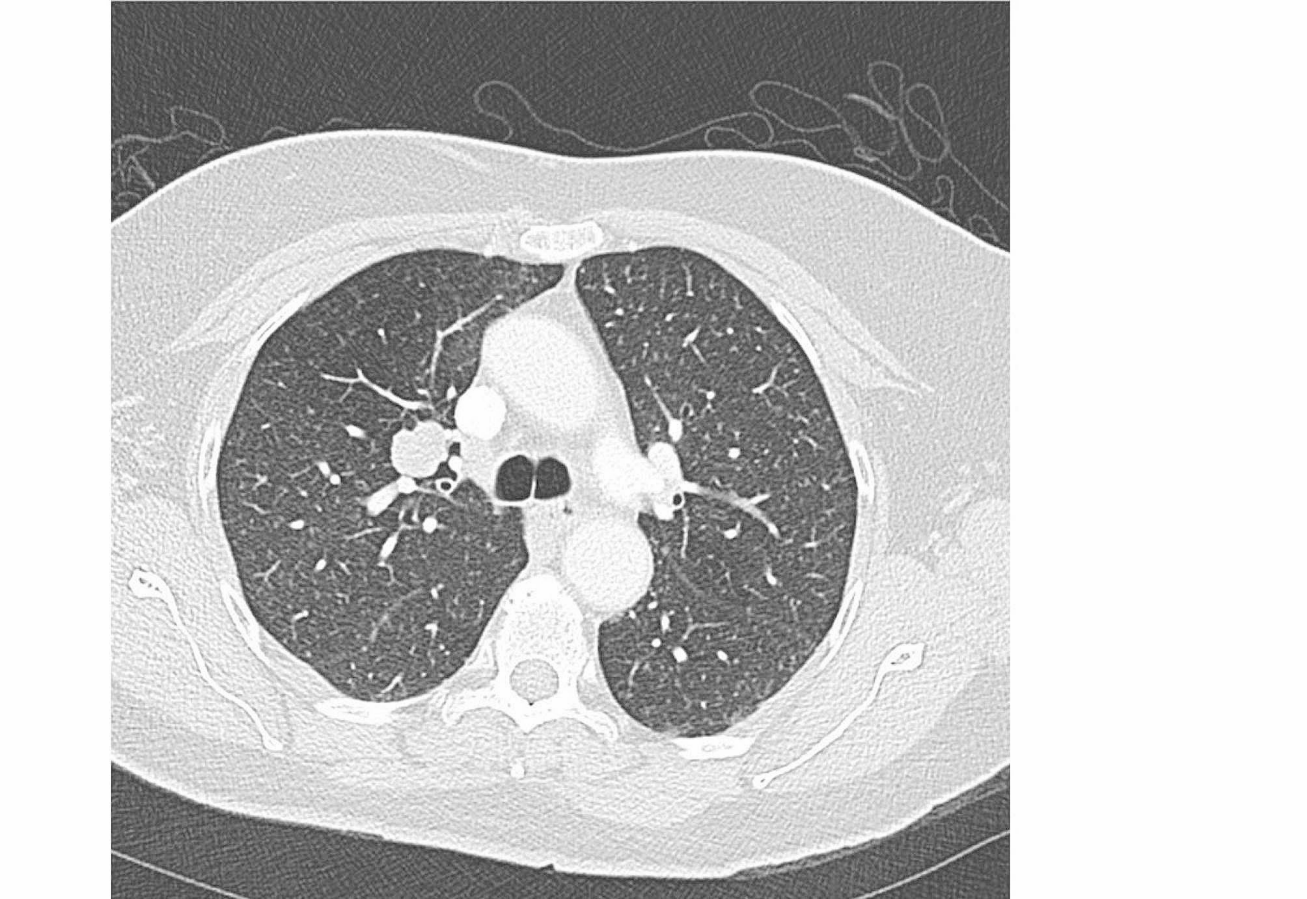
CT scan showing a mass with a dominant 2.1 × 1.7 cm solid component

**Fig. 2:**
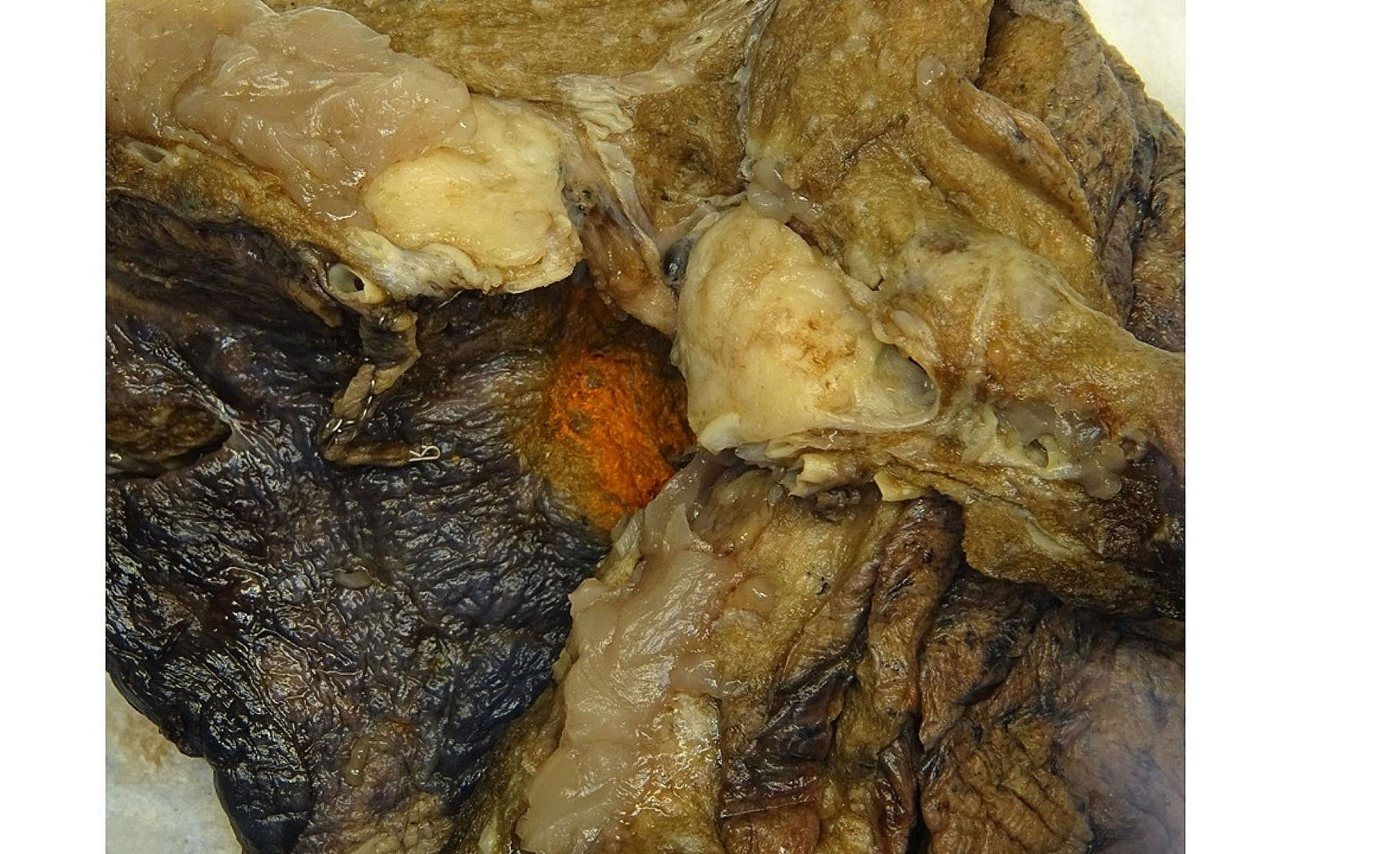
Gross photograph of the lobe resection, demonstrating a 1.7 × 1.5 × 1.5 cm white, bulging mass that was abutting the pleural surface

**Fig. 3:**
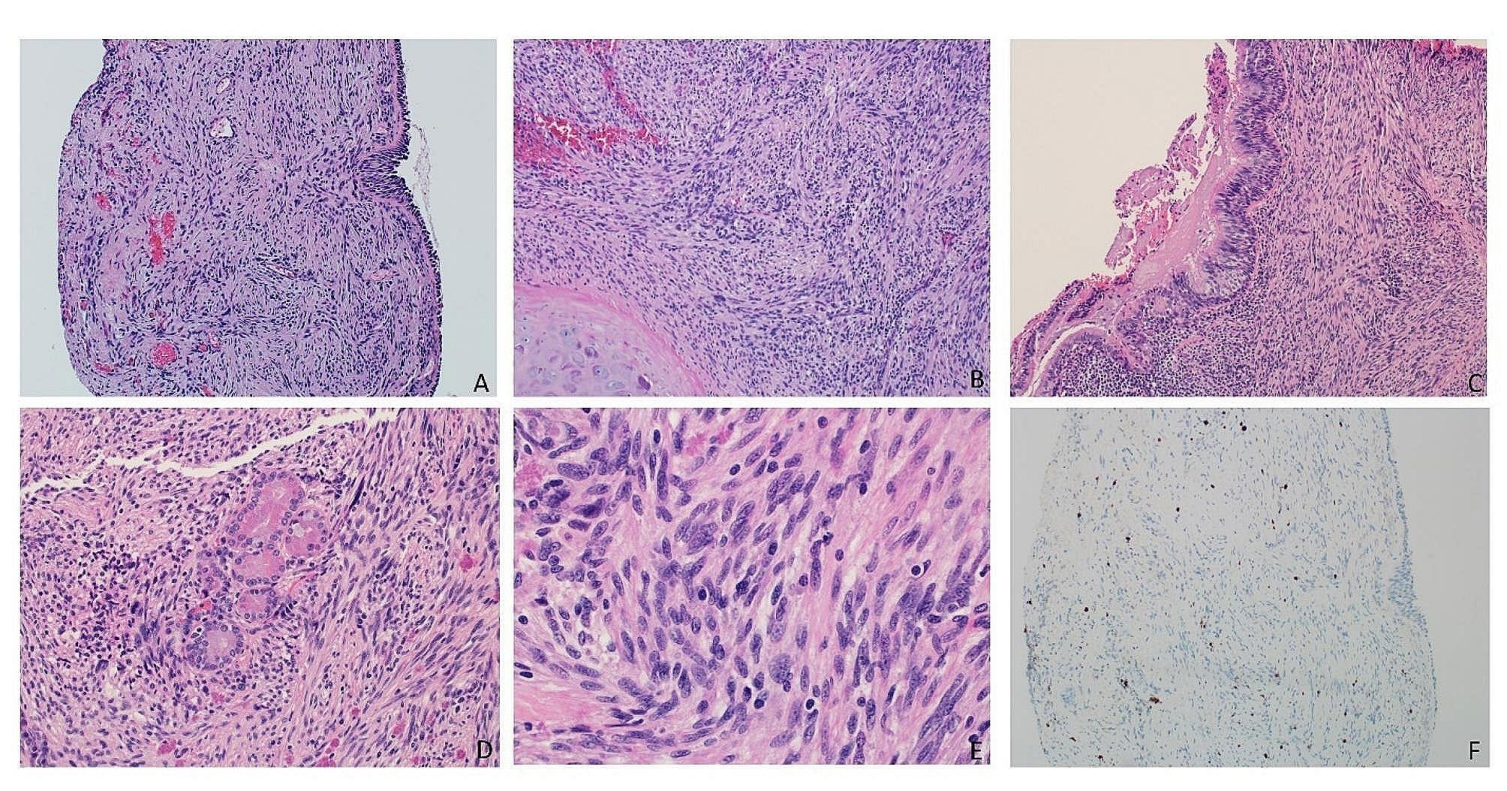
**A)** Hematoxylin and eosin stained slide showing whorls and fascicles of primitive and monomorphic spindle cells (10x); **B)** Hematoxylin and eosin stained slide showing adjacent respiratory cartilage (10x), **C)** adjacent respiratory epithelium (10x), **D)** entrapped salivary gland-type tissue (20x), and **E)** high-power showing nuclear pleomorphism (40x); **F)** low proliferative index highlighted by Ki-67 (10x)

**Fig. 4:**
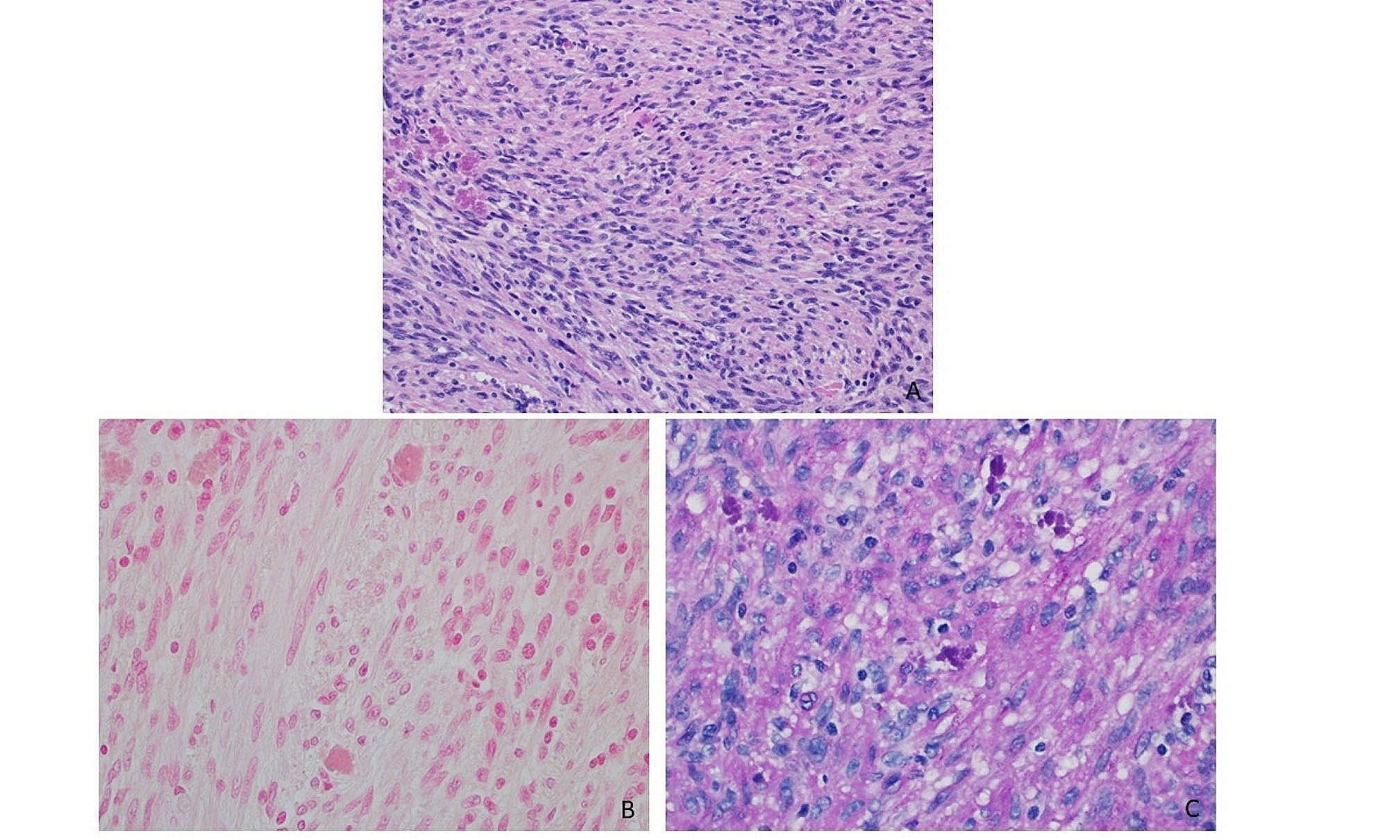
Hematoxylin and eosin-stained slide (20x magnification) showing crystalline substance within the tumor that is B) negative for VonKassa iron stain and C) positive for periodic acid Schiff (PAS) stain

A 74-year-old woman with a 40-year smoking history was found to have a right upper lobe lung nodule that was discovered on routine surveillance computed tomography (CT) scan. The imaging studies described a mass with a dominant 2.1 × 1.7 cm solid component (Fig. 1). The patient underwent an endobronchial biopsy of the right upper lung mass which revealed a spindle cell tumor in the bronchial submucosa with minimal variability in nuclear size arranged in whorls and fascicles with very rare mitotic activity. A preliminary diagnosis of low-grade spindle cell neoplasm was provided and the patient was treated with lobectomy to obtain a negative margin. The lobe was received intact, and sectioning revealed a 1.7 × 1.5 × 1.5 cm white, bulging mass that was abutting the pleural surface (Fig. 2). Histologic sections of the entire tumor recapitulated biopsy findings, showing whorls and fascicles of primitive and monomorphic spindle cells with minimal mitotic activity (< 1 per 10 high-powered fields) and no necrosis (Fig. 3). On high-power magnification, the tumor cells showed very mild nuclear size variety and occasional overlap with ovoid to cigar-shaped nuclei, inconspicuous nucleoli, and ample eosinophilic cytoplasm with indistinct cell borders. The tumor originated in the bronchial wall and showed an infiltrative pattern surrounding bronchial cartilage and submucosal glands. Focal ulceration into the bronchial mucosa was also noted. A panel of immunohistochemical stains was performed which revealed patchy positive staining for smooth muscle actin (SMA), and negative staining for cytokeratins (CK AE1/AE3, CK8/18), S100, CD34, ALK-1, desmin, myoD1, and myogenin. The proliferative rate was relatively low, with a Ki-67 of ~ 5%. A peculiar previously undescribed finding was the presence of scattered periodic acid Schiff (PAS) positive crystalloid structures located throughout the tumor (Fig. 4). These are of unclear origin but may represent entrapped concretions of normal submucosal gland secretions or material produced by the tumor.

**Fig. 5:**
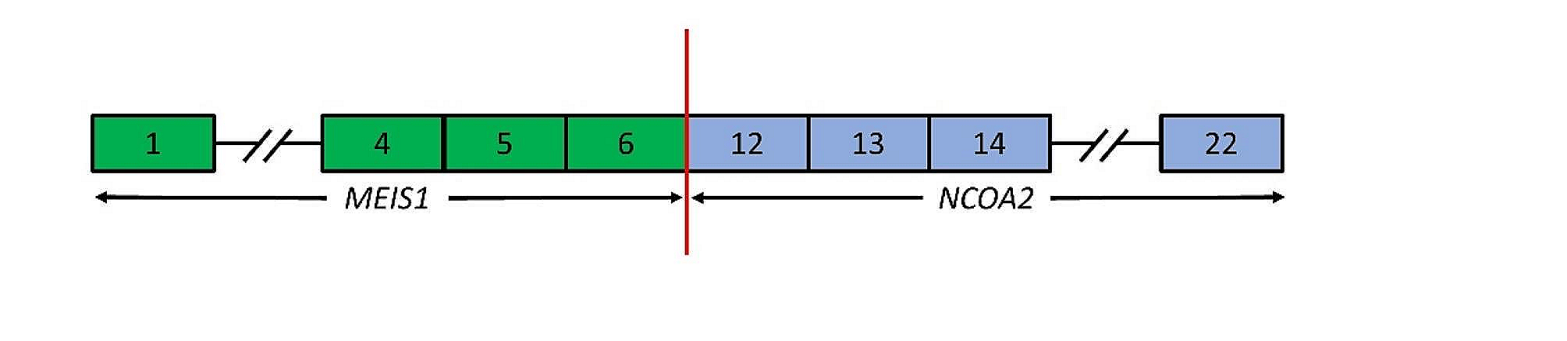
Diagrammatic representation of the fusion identified in this case

Molecular testing was performed using the PGDx elio tissue complete panel (Labcorp) and the Archer FusionPlex panel (IDT) for assessment of sequence variants and rearrangements. No variants of known or possible clinical significance were found, and the tumor was shown to be microsatellite stable with a mutational burden of 4.6 mutations per megabase. The fusion panel revealed an in-frame fusion of *MEIS1* (Chr2: 66,670,180) and *NCOA2* (Chr8: 71,060,718) (Fig. 3) in 267 reads using genome assembly GRCh37/hg19 resulting in a fusion of *MEIS1* exon 6 to *NCOA2* exon 12 (*MEIS1* NM_002398.3, *NCOA2* NM_001321703.2) (Fig. 5). Additional imaging studies, including CT and PET scans, were performed and showed no other evidence of disease. Taken together, the imaging, histologic, immunohistochemical, and molecular findings led to a diagnosis of a primary lung low-grade undifferentiated spindle cell sarcoma with *MEIS1::NCOA2* fusion. Continued follow-up from diagnosis to present (2 years) has still revealed no additional site of primary and no disease recurrence.

## Discussion and conclusions

The *NCOA2* gene (nuclear receptor co-activator 2) is located on chromosome band 8q13 and encodes a transcriptional coactivator for nuclear hormone receptors that include steroid, thyroid, retinoid, and vitamin D receptors [4]. *NCOA2* has been described as a 3’ partner in a number of fusion-associated sarcomas including mesenchymal chondrosarcoma (*HEY1::NCOA2*), spindle cell rhabdomyosarcoma (*VGLL2::NCOA2*, *SRF::NCOA2*, or *TEAD1::NCOA2*) [5], and soft tissue angiofibroma (*AHRR::NCOA2*) [6, 7].In these settings, the *NCOA2* fusion gene typically retains its C-terminal transcription activation domains 1 and 2, which leads to the prevailing theory that *NCOA2* is targeted by a DNA-binding domain that is provided by the N-terminal partner gene [8].

The role of *MEIS1* as a fusion partner in fusion-associated sarcomas is less well understood. The *MEIS1* gene, located at chromosome 2p14, encodes a protein within the three amino acid loop extension (TALE) homeodomain transcription factor family which is thought to function through the activation of Hox transcription factors [9]. The *MEIS1* gene is postulated to have key roles in cardiac regeneration and stem cell function, as well as potential for tumorigenesis [10, 11]. Interestingly, *MEIS1* is known, in isolation, to function as both a tumor suppressor gene as well as an oncogene in several tumor types [12–16]. Aside from *NCOA2* fusions in the current entity and a subset of spindle cell rhabdomyosarcomas, *MEIS1* has not been associated with gene fusion related cancers, though overexpression has been associated with myeloid leukemias [1]. The common partners for Low-grade undifferentiated spindle cell sarcomas with *MEIS1::NCOA1/2* fusions represent a recently described group of lesions initially presented in a series of two patients with primitive-appearing spindle cell sarcomas with non-specific immunohistochemical profiles found in the kidneys [1]. Moreover, a number of other reports have been published that demonstrated these tumors occurring in the kidney, uterine corpus, vagina, scrotum, and para-rectal area [2, 3]. Previous studies have noted that the broad expression of *MEIS1* across numerous human tissue types seemed to be at odds with the relative consistent localization of these tumors to the genitourinary tract [1], and this case of a lung primary seems to support the potential for numerous body sites to give rise to these tumors.

The diagnosis of *MEIS1::NCOA2* fused undifferentiated sarcomas can provide significant diagnostic challenges depending on the site of origin. A low-grade appearing spindle cell neoplasm in the lung raises the differential diagnosis of leiomyoma (either primary or benign metastasizing), metastatic low-grade appearing leiomyosarcoma, solitary fibrous tumors, schwannoma, and other low-grade metastatic spindle cell neoplasms. Some of this differential diagnosis can be investigated with a combination of immunohistochemical stains, diligent historical chart review, and imaging studies.

The overall behavior of these lesions is not well established, given the paucity of long-term follow up and rarity, but the current data seems to suggest a “low-grade” clinical course with rare reports of distant metastases (kidney to the lung), and local aggressiveness. More data is needed to fully characterize this lesion [3, 17]. The current case represents the first reported case of this entity in a solitary, central lung mass with no other sites of origin seen on imaging to suggest metastasis. This unique case broadens the perspective on *MEIS1::NCOA2*-rearranged sarcomas and further workup for this rearrangement should be considered in the appropriate setting regardless of location.

## Data Availability

Data sharing is not applicable to this article as no datasets were generated or analyzed during the current study.

## Declarations

### Author’s information

FO is associate professor of pathology at Northwestern University and is a fellowship-trained bone and soft tissue pathologist.
